# Multi-disciplinary management of recurrent gastrointestinal stromal tumor harboring KIT exon 11 mutation with the switch-control kinase inhibitor ripretinib and surgery

**DOI:** 10.18632/oncoscience.586

**Published:** 2023-09-20

**Authors:** Mohamed A. Gouda, Filip Janku, Neeta Somaiah, Kelly K. Hunt, Sireesha Yedururi, Vivek Subbiah

**Affiliations:** ^1^Department of Investigational Cancer Therapeutics, The University of Texas MD Anderson Cancer Center, Houston, TX 77030, USA; ^2^Department of Sarcoma Medical Oncology, The University of Texas MD Anderson Cancer Center, Houston, TX 77030, USA; ^3^Departments of Breast Surgical Oncology and Surgical Oncology, The University of Texas MD Anderson Cancer Center, Houston, TX 77030, USA; ^4^Department of Abdominal Imaging, The University of Texas MD Anderson Cancer Center, Houston, TX 77030, USA; ^5^Sarah Cannon Research Institute, Nashville, TN 37203, USA

**Keywords:** ripretinib, precision oncology, sarcoma, surgery

## Abstract

Ripretinib is a tyrosine kinase inhibitor that was approved by the United States FDA in 2020 for treatment of advanced gastrointestinal stromal tumor (GIST) in patients who received prior treatment with three or more tyrosine kinase inhibitors. In this case report, we show the durable clinical benefit achieved in a patient with GIST by using ripretinib and repeated timely surgical resection of limited disease progression. The total time on ripretinib was 43 months which is longer than the current reported data from ripretinib clinical trials. Such approach for using multi-disciplinary disease management can improve the durability of response to tyrosine kinase inhibitors, including ripretinib, and associated clinical outcomes.

## INTRODUCTION

Gastrointestinal Stromal Tumor (GIST) is the most common mesenchymal tumor of the gastrointestinal tract [1]. Most cases of GIST harbor a mutation in either *KIT* or *PDGFRA* gene [2–4]. Use of targeted therapies has revolutionized treatment of GIST starting with imatinib in 2002 which has controlled tumor growth in around 80% of patients [5]. Tyrosine kinase inhibitors (TKIs) such as sunitinib and regorafenib are used post-imatinib and have conferred modest clinical benefit [6–8]. However, recurrent GIST post imatinib has remained a challenge even with newer agents and multiple combination therapies.

Ripretinib is a novel TKI that was FDA approved in 2020 for treatment of advanced GIST in patients who received prior treatment with three or more TKIs, including imatinib [9]. Ripretinib is a “switch-control” kinase inhibitor that forces the activation loop (or activation “switch”) into an inactive conformation and inhibits broad spectrum of *KIT* and *PDGFRA* mutant variants including activation loop mutations [10]. In addition to an acceptable safety profile, ripretinib has been shown in the INVICTUS randomized phase 3 trial (NCT03353753) to improve the median progression-free survival (PFS) and overall survival (OS) compared to placebo control (6.3 versus 1 month for PFS; and 18.2 vs. 6.3 months for OS) [11, 12]. Herein, we describe the use of ripretinib in a patient with recurrent GIST harboring a *KIT* exon 11 mutation following treatment with multiple TKIs. The patient was managed with ripretinib and surgical resection of progressing lesions at multiple time points which led to extended clinical benefit.

## CASE PRESENTATION

A Hispanic female in her late 20s presented with left lower quadrant pain initially attributed to an ovarian cyst. A workup with computed tomography (CT) evaluation, revealed an incidental 6 cm duodenal mass with central necrosis. She underwent exploratory laparotomy with resection of the mass which was diagnosed as GIST with high malignant potential and positive immunohistochemistry for c-KIT. She was followed with close observation for 3 years before a recurrence was noted in the surgical bed in CT scans. The patient received a pre-operative 5-day course of imatinib as part of a clinical trial followed by surgery and adjuvant imatinib (600 mg daily) for 2 years.

Nearly two years after stopping imatinib, surveillance CT imaging demonstrated local recurrence and left retroperitoneal lymphadenopathy. Patient was restarted on imatinib (400 mg daily) and had disease control for four more years, until restaging scans showed progressing disease in paraaortic lymph node and surgical bed. The imatinib dose was increased to 800 mg daily but ultimately failed to control tumor growth, and the patient experienced disease progression. Treatment was switched to sunitinib (37.5 mg daily), a multi-kinase inhibitor with activity against PDGFR and c-KIT, with subsequent disease control. A year later, her tumor showed mixed response while on sunitinib and a multi-disciplinary tumor board recommended surgical resection of progressing lesions. Patient underwent surgical resection and after surgery she continued sunitinib for two years until she developed disease progression in abdominal lymph nodes.

**Figure 1:**
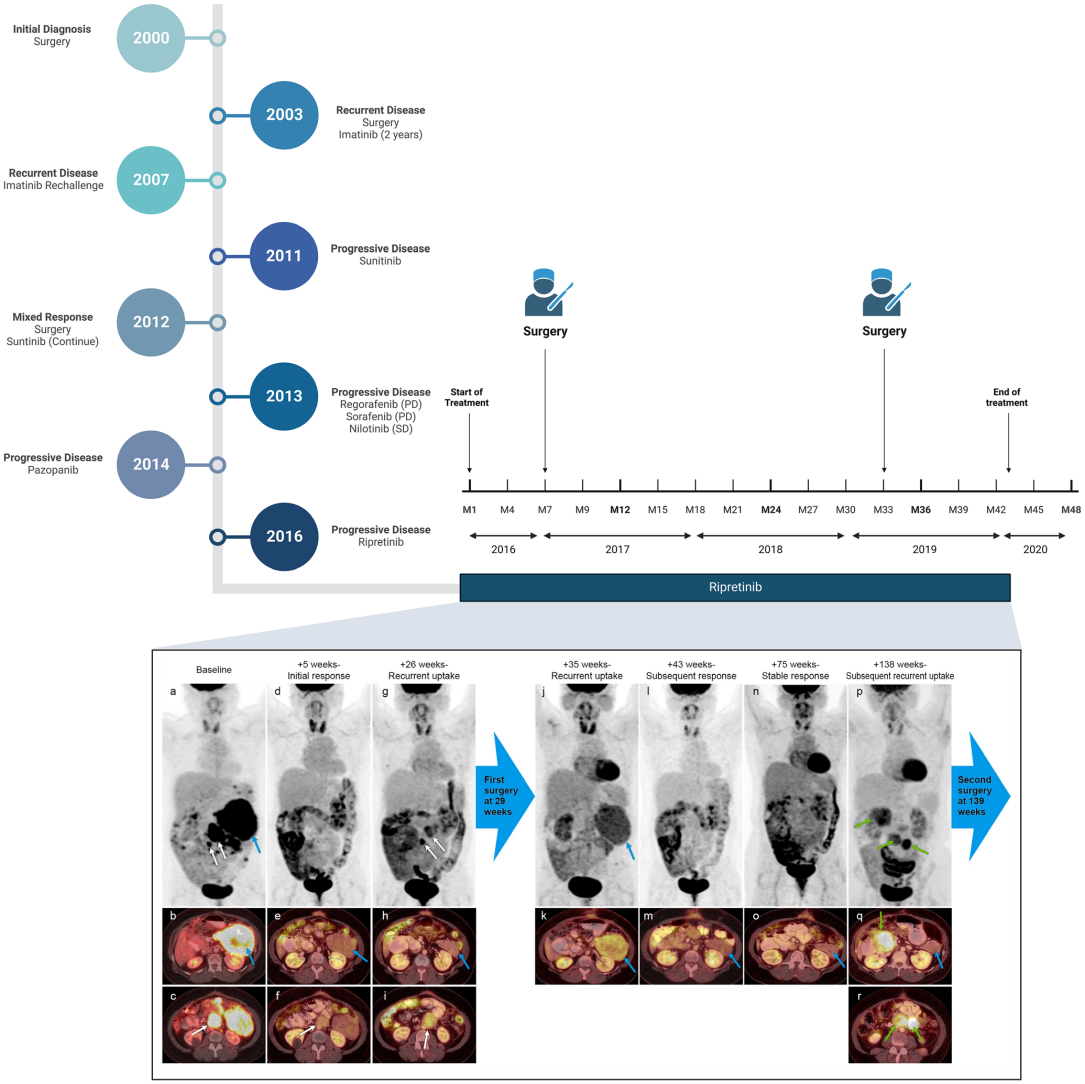
Clinical history of the patient and PET/CT images of patient while on Ripretinib therapy. Representative serial PET/CT imaging studies from baseline (prior to initiation of ripretinib) to immediately before her second surgery are illustrated in the figure. The MIP images from PET are illustrated in the top row and fused PET/CT images are illustrated in subsequent rows. The baseline PET/CT (**a**–**c**) shows multiple FDG-avid peritoneal/retroperitoneal implants of which 3 target areas are annotated (dominant lesion in the left upper quadrant is annotated with blue arrow and two smaller lesions are annotated with white arrows). Follow-up PET/CT at 5 weeks (**d**–**f**) shows decreased size and uptake of all implants including the target lesions. Follow up PET/CT at 26 weeks (**g**–**i**) shows recurrent uptake in two smaller target lesions near the midline (white arrows) and persistent response in the dominant lesion in the left upper quadrant (blue arrow). Only one of the two smaller lesions is illustrated with axial fused PET/CT images c, f and i. These two smaller lesions showing recurrent uptake were resected at ~29 weeks. Follow-up PET/CT images after initial surgery and 35 weeks from baseline (**j**, **k**) showed recurrent uptake in the previously responding dominant left upper quadrant implant (blue arrows), which resolved on the subsequent follow-up PET/CTs at 43 weeks (**l**, **m**) and 75 weeks (**n**, **o**). The disease then slowly progressed over the next few studies with eventual development of 3 new areas of uptake illustrated on 138 week follow-up PET/CT (**p**–**r**). The new sites of uptake are annotated with green arrows. Note that the dominant left upper quadrant implant shows persistent response at the 138 week follow-up study (blue arrow). The patient then underwent second surgical resection and subsequent follow-up imaging (not illustrated here) showed residual disease that further responded to treatment and then progressed with development of new liver metastases.

The patient was started on regorafenib (120 mg), a multi-kinase inhibitor of VEGFR1-3, PDGFRB, FGFR1, RAF and KIT, and subsequently sorafenib (800 mg daily), a multi-kinase inhibitor of VEGF, PDGFRB, and KIT, but neither drugs was able to control her disease (best response was progressive disease). She was initiated on nilotinib (800 mg daily) and continued treatment for one year when her scans started to show evidence of disease progression. Pazopanib was administered for two years with partial response but unfortunately her disease progressed (Figure 1).

At this time, the patient presented to the phase I program to explore the options of enrollment in a clinical trial. Based on the tumor *KIT* alteration, she was enrolled on investigational treatment with ripretinib (DCC-2618; NCT02571036). Baseline PET/CT showed involvement of multiple peritoneal implants and abdominal lymph nodes. Following treatment with ripretinib, initial re-staging with PET/CT scans showed partial response (−34% change in target lesions per RECIST) that was associated with clinical benefit in the form of resolving abdominal pain and subjective feeling of clinical improvement. Four months later, scans showed progressive disease in two smaller lesions with stable disease in the dominant target lesions. Given the clinical benefit and subjective improvement of pain, non-responding lesions were surgically resected. After surgery, patient was re-initiated on ripretinib and achieved additional shrinkage resulting in partial response (−32% change in target lesions). Response to ripretinib was maintained for 26 months until she developed progression in target lesions. Over the following few months, dose escalation from 100 mg BID to 150 mg BID was performed, as specified on the trial to control increase in the size of target lesions. This Intrapatient dose escalation resulted in clinical benefit in the form of improved pain and quality of life. However, eventually, the patient developed anemia and gastrointestinal bleeding that was attributed to development of a large fungating duodenal mass. She underwent surgical resection of the mass and then she continued treatment with ripretinib 150 mg BID given the lack of further systemic treatment options. Tissue next-generation sequencing (NGS) by that time confirmed the presence of *KIT* exon 11 p.N567_Q575delinsKEV (compared to retrospective analysis of older tissue blocks that showed both *KIT* exon 13 N665T and *KIT* exon 11 p.N567_Q575delinsKEV). Patient continued treatment for another 10 months when her CT scans, unfortunately, showed disease progression. Liquid biopsy NGS at time of progression showed the same mutational profile of *KIT* exon 11 p.N567_Q575delinsKEV. Patient was taken off ripretinib and enrolled onto a clinical trial with another investigational agent (Figure 1).

## DISCUSSION

Recurrent/relapsed GIST is a clinically challenging disease. Herein, we present a report of successful disease control of GIST (associated with *KIT* exon 11 mutation) utilizing treatment with the novel switch-kinase inhibitor ripretinib alternating with surgery to control resistant/non-responding lesions. Current data on ripretinib come from the INVICTUS randomized controlled trial which suggested a median PFS of 6.3 months (95% CI 4.6–6.9) for patients receiving active treatment. In the phase 1 trial in which patient was enrolled, the study yielded a confirmed objective response rate (ORR) of 11.3% (*n* = 16/142). This rate varied from 7.2% (*n* = 6/83; fourth line or greater) to 19.4% (*n* = 6/31; second line) depending on the treatment line. Additionally, the median PFS ranged from 5.5 months (fourth line or greater) to 10.7 months (second line), as assessed by the study investigators [13]. In our patient, the total time on therapy with ripretinib was 43 months with the use of timely surgery for limited progression. Similar strategy of using surgery for limited progression or residual disease is often used with imatinib and is supportive by multiple retrospective reports.

Following progression on multiple TKIs (including imatinib, sunitinib, regorafenib, sorafenib, nilotinib, and pazopanib), our patient started treatment with ripretinib, and continued treatment as restaging scans showed partial response or stable disease with clinical benefit. Ripretinib has an inhibitory effect on *KIT* tyrosine kinase which was part of this patient’s tumor mutational profile (*KIT* exon 13 N655T and *KIT* exon 11 p.N567_Q575delinsKEV). It was approved by the FDA in 2020 after data from the INVICTUS international multicenter placebo-controlled phase 3 trial which showed a greater objective response rate as well as markedly improved median PFS compared to placebo [11].

Dose escalation has been used at some point in our patient, as a tool to overcome resistance, and led to control of progressing disease. Adaptive and evasive resistance with TKIs has been previously reported with multiple drugs [14–17]. Such resistance due to decreased sensitivity to TKIs can be overcome through intrapatient dose escalation. For example, in patients treated with osimertinib for *EGFR* mutated non-small cell lung cancer (NSCLC), intracranial and leptomeningeal progression was controlled by increasing the dose from 80 mg to 160 mg QD in anecdotal studies [18–20]. In fact, dose escalation to overcome disease progression has been reported in GIST with other TKIs [21] and also with ripretinib [22, 23].

Another mechanism of resistance for TKIs is the development of novel mutations. For example, additional mutations in *KIT* and *PDGFRA* were detected post-treatment in patients with GIST progressing on TKI therapy [24]. The clonal evolution of cancer can be challenging particularly with multi-kinase inhibitors since it can lead to isolated subclonal progression in heterogenous tumor sites [25–28]. Such localized progression, as seen in our patient, can be managed through surgical resection of non-responding lesions [29, 30]. Surgical management of isolated progression was performed twice during the course of treatment with ripretinib in our patient and led to a prolonged clinical benefit.

Investigational therapy with targeted therapeutic agents should allow intra-patient dose escalation, treatment beyond progression for clinical benefit, and local control of non-target lesions especially if the patient is deriving clinical benefit. Multi-disciplinary care with surgery and targeted therapy can extend duration on treatment with ripretinib.
